# Risk Behaviors in Teens with Chronic Kidney Disease: A Study from the Midwest Pediatric Nephrology Consortium

**DOI:** 10.1155/2019/7828406

**Published:** 2019-12-04

**Authors:** Nianzhou Xiao, Adrienne Stolfi, Rossana Malatesta-Muncher, Reshma Bholah, Amy Kogon, Angelica Eddington, Deepa Chand, Larry A. Greenbaum, Coral Hanevold, Cheryl L Tran, Aftab Chishti, Keefe Davis, Robyn Matloff, Robert Woroniecki, Colleen Klosterman, Kera Luckritz, Abiodun Omoloja

**Affiliations:** ^1^Pediatric Nephrology, Valley Children's Healthcare, Madera, CA, USA; ^2^Department of Pediatrics, Wright State University, Dayton, OH, USA; ^3^Division of Nephrology, Texas Children's Hospital, Houston, TX, USA; ^4^Pediatric Nephrology, Children's Hospital of Richmond at VCU, Richmond, VA, USA; ^5^Division of Nephrology, Children's Hospital of Philadelphia, Philadelphia, PA, USA; ^6^Department of Pediatrics, University of Tennessee Health Sciences Center, Memphis, TN, USA; ^7^AbbVie, Chicago, IL, USA; ^8^Division of Pediatric Nephrology, Emory University School of Medicine and Children's Healthcare of Atlanta, Atlanta, GA, USA; ^9^Division of Nephrology, Seattle Children's Hospital, Seattle, WA, USA; ^10^Division of Pediatric Nephrology, Mayo Clinic, Rochester, MN, USA; ^11^Division of Nephrology Kentucky Children's Hospital, Lexington, KY, USA; ^12^St. Louis Children's Hospital, St. Louis, MO, USA; ^13^Division of Nephrology, Connecticut Children's Medical Center, Hartford, CT, USA; ^14^Division of Nephrology and Hypertension, Stony Brook Children's Hospital, Stony Brook, NY, USA; ^15^Division of Nephrology and Hypertension, Cincinnati Children's Hospital Medical Center, Cincinnati, OH, USA; ^16^C.S. Mott Children's Hospital, Ann Arbor, MI, USA

## Abstract

**Introduction:**

There is a paucity of information about risk behaviors in adolescents with chronic kidney disease (CKD). We designed this study to assess the prevalence of risk behaviors among teens with CKD in the United States and to investigate any associations between risk behavior and patient or disease characteristics.

**Methods:**

After informed consent, adolescents with CKD completed an anonymous, confidential, electronic web-based questionnaire to measure risk behaviors within five domains: sex, teen driving, alcohol and tobacco consumption, illicit drug use, and depression-related risk behavior. The reference group was composed of age-, gender-, and race-matched US high school students.

**Results:**

When compared with controls, teens with CKD showed significantly lower prevalence of risk behaviors, except for similar use of alcohol or illicit substances during sex (22.5% vs. 20.8%, *p*=0.71), feeling depressed for ≥2 weeks (24.3% vs. 29.1%, *p*=0.07), and suicide attempt resulting in injury needing medical attention (36.4% vs. 32.5%, *p*=0.78). Furthermore, the CKD group had low risk perception of cigarettes (28%), alcohol (34%), marijuana (50%), and illicit prescription drug (28%). Use of two or more substances was significantly associated with depression and suicidal attempts (*p* < 0.05) among teens with CKD.

**Conclusions:**

Teens with CKD showed significantly lower prevalence of risk behaviors than controls. Certain patient characteristics were associated with increased risk behaviors among the CKD group. These data are somewhat reassuring, but children with CKD still need routine assessment of and counselling about risk behaviors.

## 1. Introduction

Risk behaviors contribute markedly to the leading causes of morbidity, mortality, and social problems among adolescents and adults in the United States (US) [1]. Risk behaviors can be defined as any behavior that compromises one's psychosocial aspects of successful adolescent development [2]. Examples of risk behaviors include the following: unprotected sexual intercourse; tobacco, alcohol, and other drug use; unhealthy dietary behaviors; inadequate physical activity; and behaviors that contribute to unintentional injuries and violence. In addition, the advent of texting, cyber bullying, and synthetic drugs has widened the scope of adolescent risk behaviors. These behaviors are often established during adolescence but continue through adulthood [1, 3].

The presence of a chronic illness can add more challenges to adolescence and further influence participation in and outcomes of risk behaviors. For instance, patients with end-stage renal disease (ESRD) may need to receive hemodialysis 3 or more times per week, which may impact academic growth, socialization, and self-esteem significantly [4, 5]. These challenges may lead them to engage in risk activities to achieve peer acceptance as well as a personal sense of independence [6]. Other studies, however, showed the opposite. Hollen et al. reported a lower prevalence of lifetime substance use in teen cancer survivors than in the general US population [7]. In addition, Valencia and Cromer studied chronically ill teens with cystic fibrosis, myelomeningocele, hemophilia, and HIV and reported lower prevalence of substance use. They suggested that perhaps teens with those particular chronic illnesses were restricted from engaging in substance use by their medical condition [8]. A study evaluating specific risk behavior [9] and studies in small and selected subjects [10] have provided important but limited insights about risk behaviors in adolescents with CKD. Nephrologists especially those who work with adolescents and young adults may not have provided routine risk behaviors counselling because of lack of comprehensive understanding of its prevalence and severity. We designed the Assessment of Risk Behavior in teens with Chronic Kidney Disease (ASK KIDD) study to establish the prevalence of risk behaviors in a large group of adolescents with CKD. We compared their data with a frequency-matched sample derived from the National 2015 Youth Risk Behavior Survey (YRBS) and determined associations with patient characteristics.

## 2. Materials and Methods

### 2.1. Study Subjects

Consecutive patients with CKD from 15 pediatric nephrology centers of the Midwest Pediatric Nephrology Consortium (MWPNC) (http://mwpnc.org/) participated in the study. Criteria for inclusion into the ASK KIDD study were children 13 to 19 years of age with at least CKD stage II (estimated glomerular filtration rate (eGFR) < 89 ml/min/1.73 m^2^) or were transplant recipients. Patients receiving dialysis were required to be on dialysis for at least 6 weeks. We excluded subjects that could not conduct the computer-based survey independently. Institutional Review Board of each participating center approved the study. Consent and assent were obtained per institutional guidelines.

### 2.2. Survey and Measures

The national Youth Risk Behavior Survey (YRBS) is an open-source survey administered biannually by the Centers for Disease Control and Prevention (CDC) since 1991 to high school students in the US by paper. The reliability of administering the YRBS survey online has been documented [11]. The study survey comprised 42 of the 99 National YRBS questions that focused on five domains: driving safety-related behaviors, sexual behaviors, substance and alcohol use, bullying and conflicts, and depression and suicidal behaviors. Four additional questions were asked regarding perceptions of the risk of harm from certain behaviors. The questions were “How much do you think people risk harming themselves if they … (1) Smoke one or more packs of cigarettes per day, (2) Take one or two drinks of an alcoholic beverage per day, (3) Smoke marijuana once or twice a week, and (4) Use prescription drugs that are not prescribed to them.” Response options for the 4 questions were no risk, slight risk, moderate risk, and great risk. The complete study survey is shown in [Supplementary-material supplementary-material-1].

An anonymous web-based survey was self-administered privately on an electronic device. No personal information was stored on the devices. Subjects were compensated for their time with a $15 gift card upon completion of the survey.

### 2.3. Statistical Analysis

Demographic variables collected in the CKD cohort included age, gender, race, CKD category (nondialysis CKD, dialysis, or transplant), and number of medications taken per day. Dialysis participants were asked which modality was used (hemodialysis or peritoneal dialysis), and duration of dialysis (<1 year, 1-2 years, or >2 years). Adolescents with kidney transplants were questioned how long they had had a transplant (<1 year, 1-2 years, or >2 years).

Descriptive statistics included frequencies and percent of nonmissing data and mean ± SD for age. Data from the 2015 YRBS National High School data set were used as the control sample. Adolescents in both data sets were stratified by age (13–15 vs. 16–19 years), gender, and race (white vs. nonwhite), resulting in eight strata. The proportion of adolescents in the ASK KIDD and YRBS samples that fell into each of the strata was then determined. To obtain the maximum possible sample size from the YRBS data set, all participants in one stratum in the YRBS data set were selected and then random sampling was conducted for the remaining 7 strata to obtain proportions that matched the proportions in the ASK KIDD sample ([Supplementary-material supplementary-material-1]). Of 15,294 adolescents in the national data set, 8530 could be used as the comparison population. Differences in proportions engaged in risk behaviors between the ASK KIDD sample and the control sample were analyzed with one-sample chi-square tests, using the control sample proportions as the hypothesized population proportions. Age at first sexual intercourse was estimated from the frequency distribution for the age in 1-year intervals, with 11 and 17 years used for the lower interval of ≤11 years and upper interval of ≥17 years. For age at first consumption of alcohol and first time smoking marijuana, the intervals for the frequency distributions were ≤8 years, 9-10, 11-12, 13-14, 15-16, and ≥17 years. Mean ages were estimated using lower and upper ages of 8 and 17 years, and the midpoints of the 2-year intervals. Approximate normal distribution of the estimated mean ages was confirmed, and one-sample *t* tests were used for comparisons to the control sample.

To analyze differences in high risk behaviors and attitudes about high risk behaviors based on age, gender, race, type of CKD, and number of medications per day (0–5 vs. 6 or more), multiple logistic regression analysis was performed with all of the above demographic variables included in the models. Adjusted odds ratios (AORs) with 95% confidence intervals (CIs) were determined. For certain high risk behaviors, fewer than 20 teens exhibited the behavior resulting in unstable estimates, so only unadjusted odds ratios (95% CI) were determined. Chi-square tests were used to compare teens with one high risk behavior vs. two or more on depression and suicide-related behaviors. Fisher's exact tests was used to compare high-risk behaviors between dialysis groups, number of years on dialysis, and number of years with a kidney transplant, owing to the small sample sizes within these groups. Response options for the 4 perception of risk of harm questions were dichotomized into no/slight risk vs. moderate/great risk and compared between age categories, gender, and race with chi-square tests.

Sample sizes varied among the variables analyzed because of the missing data for some participants. In the YRBS data set, missing responses result from skipped questions or illogical/invalid responses. Since the missing data could not be characterized as to type, no multiple imputation methods were utilized. For all analyses, *p* values ≤0.05 were considered statistically significant. All analyses were performed with IBM® SPSS® Statistics for Windows, version 24 (IBM Corp, Armonk, NY).

## 3. Results

### 3.1. Study Population

Three hundred eighteen subjects from 15 United State sites completed the survey. Nine of 18 surveys from a single center were excluded because they accidently used a mock “practice” survey rather than the actual survey assigned to their site. Refusal to participate was reported by 5 centers regarding 21 subjects for various reasons, including not having enough time, not being interested, or parents not wanting the child to answer the survey questions. Refusal rates were not noted for 10 out of 15 centers. Of the 309 surveys included in the analyses, 7 had missing responses for some questions.

Slightly more males (57.0%) and older teens (age 16–19 years, 62.1%) participated in ASK KIDD (Table 1). Overall, the proportions in the eight strata based on age, gender, and race ranged from 7.8% to 21.0% ([Supplementary-material supplementary-material-1]). The majority of the CKD participants (88.1%) attended school; the remainder had graduated (9.1%) or were home schooled (4.3%). All of the adolescents in the control sample attended high schools.

### 3.2. Driving Safety-Related Behaviors

Four survey questions were designed for driving safety-related behaviors (Table 2). There was significantly less driving-related risk behaviors among teens with CKD than in the controls. In the CKD group, males were 2 times more likely to drive without seatbelt (*p*=0.006) or text during driving (*p*=0.05) than females. However, there was no such difference when comparing by age (16–19 vs. 13–15), race (white vs. nonwhite) (see Table 3), CKD category (dialysis vs. nondialysis CKD, transplant vs. nontransplant CKD), or medication burden (≥6 vs. 0–5 medications/day).

### 3.3. Sexual Experience

Five survey questions were related to sexual experience. As shown in Tables 2 and 4, fewer CKD participants reported ever having sex; and they became sexually active at a later age than the controls (26.7% vs. 41.6%, *p* < 0.001; mean ± SD 15.1 ± 1.6 vs. 14.6 ± 1.6, *p*=0.01, respectively). The percentage of participants having ≥2 partners and/or engaging in unprotected sexual intercourse, or using alcohol or illicit drug during sex were comparable in the two groups (*p*=0.065, *p*=0.708, respectively). Among those who were sexually active, condoms were the most commonly reported contraception method in the CKD and reference groups (54.8% vs. 60.2%, *p*=0.345).

Table 3 and further analysis showed that younger age and being white were associated with fewer high risk sexual behaviors in the CKD group. CKD category (dialysis vs. nondialysis CKD) and medication burden (≥6 vs. 0–5 medications/day) did not impact sexual risk behavior. Of note, transplant recipients were 3.3 times more likely than the other CKD teens to use alcohol or illicit drugs during sex (*p*=0.034).

### 3.4. Substance Use

A total of twenty survey questions addressed substance-related risk behaviors including alcohol, cigarettes, marijuana, and illicit/prescription drugs use (Tables 2 and 3). Teens with CKD reported significantly less substance use across all surveyed substances, lower incidents of being offered/sold/given illegal drugs on school property, and a later age to first alcoholic drink (mean ± SD 14.1 ± 2.3 vs. 13.3 ± 2.6, *p*=0.007) than the controls. Among those who smoked cigarettes, the CKD group preferred nicotine cigarettes over e-cigarettes, which is in contrast to the reference group (*p* < 0.001). Age first smoked marijuana was comparable between the two groups (mean ± SD 13.8 ± 2.4 vs. 13.8 ± 2.1, *p*=0.988).

Among teens with CKD, older age was associated with more risk behavior related to substance use (ever used alcohol, smoked cigarettes, used marijuana and illicit drugs in past 30 days, or was offered/sold/given an illegal drug on school property in past 12 months) (Table 3). Race, gender, and dialysis status (on vs. not on dialysis) had no impact on substance use. Transplant patients reported being offered/sold/given an illegal drug on school property more often (OR 3.5, *p*=0.02) but had fewer incidents of lifetime exposure to 1 or more illicit drugs (OR 0.4, *p*=0.038) than teens with nontransplant CKD; medication burden (≥6 medications per day) was associated with fewer drinks of alcohol in the past 30 days (OR 0.3, *p*=0.007).

Four non-YRBS survey questions were designed to address attitudes toward smoking, alcohol, marijuana, and prescription drug use. Data from the questions revealed slight to no-risk perception in 28.5%, 34.5%, 50.0%, and 28.5% of teens with CKD for smoking, alcohol, marijuana, and prescription drug use, respectively (Table 5). Males were more likely to consider substance use as having no/slight risk for smoking (33.3% vs. 22.1%, *p*=0.033), alcohol use (39.2% vs. 28.2%, *p*=0.047), and prescription drug use (29.2% vs. 13.7%, *p*=0.001). Nonwhite participants responded no risk or slight risk at a higher rate compared with white participants for smoking (37.4% vs. 21.6%, *p*=0.003), marijuana use (61.1% vs. 41.5%, *p* < 0.001), and prescription drug use (31.3% vs. 15.8%, *p* < 0.001). Fifty-five percent of the older teens considered marijuana use to be of no risk or slight risk compared with 41.7% of younger teens (*p*=0.024). There were no other differences between gender, race, and age groups for the 4 risk of harm questions.

### 3.5. Bullying and Conflicts

Table 2 shows comparison of the prevalence of physical fights (15.5% vs. 22.3%, *p*=0.004), school bullying (12.3% vs. 19.7%, *p*=0.002), and electronic bullying (6.9% vs. 14.0%, *p* < 0.001) between the groups. Among the CKD participants, age, CKD category, and medication burden did not appear to be associated with reported bullying and conflicts (*p* > 0.05). Females, however, were 2.5 times more likely to be electronically bullied than males (*p*=0.04).

### 3.6. Depression and Suicide-Related Behaviors

Among the CKD subjects, 74 of 304 participants (24.3%) reported feeling depressed for ≥2 weeks over 12 months, which was less than in controls (2459 of 8451, 29.1%) and approached significance (*p*=0.068). Overall, the CKD group reported significantly fewer seriously considered/planned/attempted suicides than the controls (11.5% vs. 17.4%, *p*=0.007), as shown in Table 2. For 4 of 11 (36.4%) participants in the CKD group who attempted suicide, the attempt resulted in an injury that needed medical attention. This was comparable to the proportion of suicide attempts that resulted in injury needing medical attention for participants in the control group (569/6784 (32.5%), *p*=0.784; Table 2).

**Figure 1:**
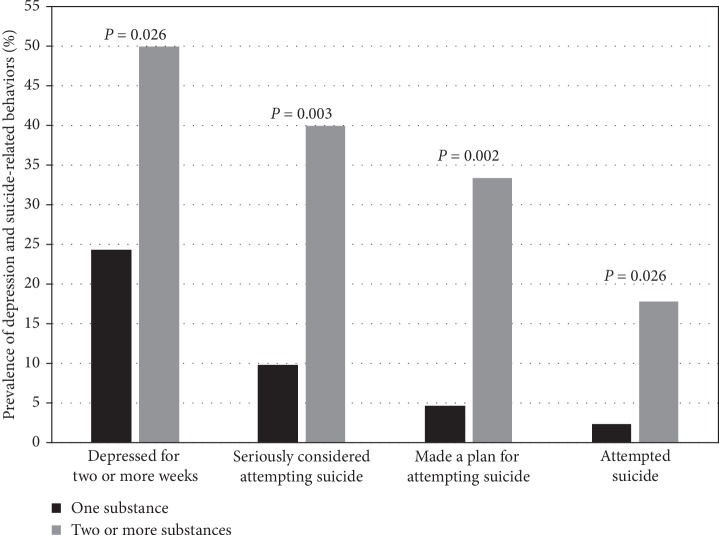
Effect of one vs. multiple risk behaviors (alcohol, smoking, marijuana, and drug use) on the prevalence of depression and suicidal behaviors in adolescents with chronic kidney disease. Values represent the percent of each high-risk behavior group with “yes” responses for depression and suicidal behaviors in the past 12 months. For the high-risk behaviors groups, sample sizes are 41 for one and 30 for two or more for the depression, considered attempting suicide, and made a suicide plan variables. For attempted suicide, sample sizes are 41 for one and 28 for two or more.

Within the CKD group, more females than males reported depressive feelings for over 2 weeks and had suicidal ideation with plan (Table 3). Age, race, CKD category, and medication burden did not impact the adjusted odd ratios for depression and suicide-related behaviors. Those who reported using two or more substances had significantly higher incidents of self-reported depression and suicide-related behaviors (*p* < 0.05, Figure 1).

## 4. Discussion and Conclusion

Risk behavior is defined as any behavior that can compromise the psychosocial aspects of successful adolescent development [2]. The ASK KIDD study sought to provide comprehensive data on risk behaviors in adolescents with CKD (CKD II-V and kidney transplant recipients) in the US. Strengths of the study lie in the large number of subjects enrolled from 15 geographically diverse centers as well as anonymity of responses. Hence, the results represent the adolescent CKD population across the US. Transplant patients appeared to be overrepresented (about 34%) in the cohort. Secondary analysis comparing transplant with nontransplant participants identified less lifetime exposure to illicit drugs or nonprescribed prescription drugs but significantly higher incidents of being offered/given/sold illegal drug among the transplant group. A possible explanation for this is that the transplant group often represents a subgroup that has been screened for family support and medical compliance among all advanced CKD patients, including those on dialysis. These factors are known to play a “protective” role against risk behaviors [12, 13], probably making them “more resistant” to illegal drugs even when offered. The transplant and nontransplant group showed no significant differences on all other studied risk behaviors.

The CKD cohort had significantly lower prevalence of risk behaviors in all five measured domains than their age-, gender-, and race-matched peers: driving safety-related behaviors, sexual behaviors, substance and alcohol use, bullying and conflicts, and depression and suicidal behaviors. This is different from the common perception that adolescents with chronic diseases have higher incidence of psychiatric symptoms and suicidal behaviors because of disease burden. Indeed, others have suggested a reduced incidence of risk behaviors in children with chronic diseases [6, 10]. In addition, our data showed no differences in incidents of self-reported depressive symptoms when correlating with medication burden or CKD category (transplant vs. nontransplant, dialysis vs. nondialysis). This is consistent with the findings of other studies that have reported that disease burden is not correlated with depressive symptoms in children [10], unlike in adults [14]. Females in our study carried higher risk of feeling depressed and attempted suicide, consistent with results reported by Kogon et al. [10]. Other depression-related findings were not fully consistent with psychiatric assessment conducted by health professionals in predialysis and dialysis patients. Bakr et al. showed that the array of psychiatric disorders did not correlate with sex, severity of anemia, or duration of hemodialysis [15]. Hernandez et al. reported similar findings of a lack of association between depression symptoms with age, time and dialysis type, or schooling [16]. A possible reason for differences is that ASK KIDD subjects were not assessed for depression using a standardized instrument as was done by Bakr et al. and Hernandez et al. in their studies. Subjects might not have recognized depression or failed to recall risk behaviors accurately leading to recall bias. Secondly, our study was bigger and included subjects with CKD stages 2–5 and not solely dialysis subjects as were in Bakr's and Hernandez's studies. ASK KIDD study raises the question of how screening for risk behaviors including depression should be carried out in this patient population, which needs to be addressed by future studies. Nevertheless, ASK KIDD provides a unique view of self-reported risk behavior and depressive symptoms from the teen perspective.

Many studies have shown that youth with chronic illness or disability are more likely to be bullied [17, 18] which is in contrast to our data. We would speculate some uncollected information may explain these findings. First, the lack of an apparent physical disability in many teens with CKD, aside from being shorter in height, may be protective of bullying over adolescents with obvious physical disabilities. Next, socioeconomic status, parental education level, and even genetic background have been reported to be associated with bullying involvement among adolescents with chronic diseases [19, 20].

Our findings should not be interpreted to mean that adolescents with CKD are well protected from risk behaviors. Considering that the use of tobacco, alcohol, marijuana, and nonprescribed prescription medications can accelerate progression of CKD [21], it is worrisome that 28% to 50% of CKD subjects did not consider that their use would cause more than mild harm to their health. Nonwhite participants were less concerned about harm from substances. This highlights the importance of discussing and educating teens about safe behaviors that should occur simultaneously with medical management, especially in the nonwhite groups. It is also important to note that while teens with CKD may have less frequent risk behaviors as a cohort, certain subgroups have exceptionally higher accumulative incidents of depression and suicidal behaviors, namely, those utilizing multiple substances. The incidents of self-reported depression and suicidal planning or attempts were 2-3 times higher than those consuming a single substance. Data also showed that the CKD group reported significantly lower incidents of feeling depressed/suicidal planning/attempted suicide than their peers. However, a larger proportion of the CKD group required medical attention after the suicide attempts. These findings suggest that risk behaviors might lead to more profound consequences in adolescents with CKD than in healthy adolescents. This might be explained by their comorbidities and healthcare-related risks. Alcohol, for instance, has been reported to be the second most common form of high risk behavior in chronically ill adolescents after smoking [22]. Consumption of any amount of alcohol below the age of 21 years is illegal in the US. Alcohol consumption is associated with judgment and cognitive impairment in the short and long term [23]. There is overwhelming evidence of negative cognitive effects from alcohol use on the developing teen brain [23–25], independent association of CKD with cognitive impairment [26, 27], and potential prescription medications interacting with alcohol and or the other substances of use [28–33]. Although prevalence of binge drinking was low (2.3%) in the ASK KIDD cohort, 8% had reported alcohol consumption in the last 30 days of the survey, mostly among the older teens and those on less than 5 medications per day. This finding was consistent with other studies of alcohol use in teens with chronic medical conditions [3]. Therefore, health providers must pay extra attention to the short- and long-term implications on the well-being of teens with CKD who consume alcohol.

Our study has several limitations: (1) The results suggest a “dose-response” of depression/suicidal behavior among CKD patients taking alcohol, smoking, marijuana, and drug. And, showed association between risk behavior and certain subject characteristics (gender, age, and disease burden for instance). However, as a cross-sectional study, it is not designed to confirm causality. (2) Additionally, there is a lack of clinical data such as laboratory results and disease progression. Future studies are warranted to assess how risk behaviors may impact health outcomes. ASK KIDD does not obtain socioeconomic variables. Transplanted participants were assumed to have better home support and were more restricted from taking risks, but the study is not designed to analyze potential correlation between detailed socioeconomic factors and those behaviors. (3) There was potential bias owing to subject selection. Patients with cognitive impairment that compromised them from participating the survey would have been excluded although they may be at risk for being impacted by psychosocial issues. We had to exclude surveys due to site-specific technical difficulties contributed to missing data. Furthermore, approximately 12% of the CKD cohort were home schooled, and thus the comparison to regular-schooled national data might be inaccurate.

Despite these limitations, the information gathered during this study can contribute to better understanding of prevalence of risk behaviors and their association with patient characteristics. In summary, risk behaviors are common in teens with CKD although occurring at lower frequencies compared with the general population. The potential consequences of risk behavior, however, could be drastic in medically complicated CKD patients. Mitigation of these behaviors could begin with a discussion with the teen beyond medical management to include education on potential negative impact of risk behaviors on their renal health outcomes. The ultimate goal is to help teens with CKD transition to adulthood safely and improve long-term outcomes.

## Figures and Tables

**Table 1:** Characteristics of adolescents with CKD.

Characteristic	*n* (%)
Age, years, mean ± SD	16.1 ± 1.8
Age	
13–15 years old	117 (37.9)
16–19 years old	192 (62.1)
Male gender	176 (57.0)
Race	
White	174 (56.3)
Black	72 (23.3)
Others	63 (20.4)
Current school attendance (*N* = 304)	
In school	268 (88.1)
Graduated	23 (7.6)
Home schooled	13 (4.3)
Kidney diseases	
Nondialysis CKD	177 (57.3)
Dialysis	28 (9.1)
Kidney transplant	104 (33.7)
Type of dialysis (*N* = 28)	
Hemodialysis	17 (60.7)
Peritoneal dialysis	11 (39.3)
Years on dialysis (*N* = 28)	
<1 year	12 (42.8)
1-2 years	8 (28.6)
>2 years	8 (28.6)
Years after kidney transplant (*N* = 104)	
<1 year	29 (27.9)
1-2 years	11 (10.6)
>2 years	64 (61.5)
Number of regular medications/day (*N* = 305)	
0–5	132 (43.3)
≥6	173 (56.7)

*N* = 309 unless otherwise specified.

**Table 2:** Comparisons of risk behaviors between adolescents with CKD (ASK KIDD) and 2015 YRBS National Data.

High risk behavior	ASK KIDD		YRBS		*P*
	*N*	*n* (%)	*N*	*n* (%)	
Driving safety-related behaviors					
Wore a seatbelt less than always	305	93 (30.5)	7508	2897 (38.6)	0.004
Past 30 d: rode with drinking driver	305	30 (9.8)	8501	1816 (21.4)	<0.001
Past 30 d: texted or emailed while driving (in teens who drove)	152	26 (17.1)	4867	1897 (39.0)	<0.001
Past 30 d: drove while drinking (in teens who drove)	152	6 (3.9)	4690	423 (9.0)	0.030

Sexual experience					
Lifetime: ever had sexual intercourse	300	80 (26.7)	7668	3189 (41.6)	<0.001
Past 3 m: ≥2 sex partners and/or no protection last time (in teens who had sexual intercourse)	80	40 (50.0)	3048	1215 (39.9)	0.065
Drank alcohol or used drugs the last time had sexual intercourse (in teens who had sexual intercourse)	80	18 (22.5)	3073	640 (20.8)	0.708

Substance abuse					
Lifetime: ever had alcohol	294	72 (24.5)	7520	4823 (64.1)	<0.001
Past 30 d: 1 or more drinks of alcohol	301	25 (8.3)	7364	2176 (29.5)	<0.001
Past 30 d: 1 or more days of binge drinking	301	7 (2.3)	7858	1188 (15.1)	<0.001
Past 30 d: smoked cigarettes or E-cigarettes	304	28 (9.2)	8104	2274 (28.1)	<0.001
Past 30 d: used marijuana	304	30 (9.9)	8351	1798 (21.5)	<0.001
Lifetime: used 1 or more illicit drugs or prescription drugs without prescription	302	42 (13.9)	6189	1645 (26.6)	<0.001
Past 12 m: someone offered, sold, or gave you an illegal drug on school property (in teens attending school)^a^	268	40 (15.0)	8121	1902 (23.4)	0.001

Bullying and conflicts					
Past 12 m: in physical fight	304	47 (15.5)	7070	1575 (22.3)	0.004
Past 12 m: bullied at school (in teens attending school)^a^	268	33 (12.3)	8451	1662 (19.7)	0.002
Past 12 m: electronically bullied	304	21 (6.9)	8459	1188 (14.0)	<0.001

Depression and suicide-related behaviors					
Past 12 m: depressed ≥2 wk	304	74 (24.3)	8451	2459 (29.1)	0.068
Past 12 m: seriously considered attempting suicide	304	35 (11.5)	8446	1472 (17.4)	0.007
Past 12 m: made a suicidal plan	304	27 (8.9)	8316	1222 (14.7)	0.004
Past 12 m: attempted suicide	299	11 (3.7)	6784	569 (8.4)	0.003
Past 12 m: suicide attempt resulted in injury needing doctor or nurse (in teens who attempted suicide)	11	4 (36.4)	569	185 (32.5)	0.784

^a^All adolescents in the YRBS dataset were attending school at the time of the survey. CKD, chronic kidney disease; d, days; m, months; wk, weeks; y, years; YRBS, Youth Risk Behavior Survey.

**Table 3:** Adjusted odds ratios (95% confidence intervals) for high-risk behaviors in the CKD group by age category, gender, and race.

High-risk behavior	*N*	Age 16–19 y (ref = 13–15 y); AOR (95% CI)	*P*	Male gender (ref = female); AOR (95% CI)	*P*	White race (ref = non-white); AOR (95% CI)	*P*
Driving safety-related behaviors							
Wore a seatbelt less than always	302	1.1 (0.7–1.9)	0.662	2.1 (1.2–3.6)	0.006	0.8 (0.5–1.4)	0.426
Past 30 d: rode with drinking driver	302	0.8 (0.4–1.7)	0.551	1.5 (0.7–3.5)	0.305	0.6 (0.3–1.3)	0.177
Past 30 d: texted or emailed while driving (in teens who drove)	152	3.6 (0.9–13.2)	0.058	2.8 (1.0–7.9)	0.050	0.5 (0.2–1.1)	0.093
Past 30 d: drove while drinking (in teens who drove)^a^	152	1.8 (0.2–16.0)	0.589	0.9 (0.2–4.3)	0.843	0.8 (0.2–4.2)	0.817

Sexual experience							
Lifetime: ever had sexual intercourse	297	10.0 (5.0–28.6)	<0.001	1.0 (0.6–2.0)	0.932	0.7 (0.4–1.1)	0.143
Past 3 m: ≥ 2 sex partners and/or no protection last time (in teens who had sexual intercourse)	78	2.5 (0.4–16.9)	0.341	1.1 (0.4–3.1)	0.731	0.3 (0.1–0.8)	0.021
Drank alcohol or used drugs last time had sexual intercourse (in teens who had sexual intercourse)^a^	78	na	na	2.4 (0.8–7.7)	0.129	1.1 (0.4–3.1)	0.901

Substance abuse							
Lifetime: ever had alcohol	289	3.3 (2.0–10.0)	<0.001	1.1 (0.6–2.0)	0.685	1.1 (0.6–2.0)	0.693
Past 30 d: 1 or more drinks of alcohol	298	2.0 (0.7–5.2)	0.171	0.7 (0.3–1.6)	0.370	0.8 (0.3–1.8)	0.535
Past 30 d: 1 or more days of binge drinking^c^	298	3.2 (0.4–28.4)	0.287	1.5 (0.3–8.4)	0.673	1.5 (0.3–8.5)	0.642
Past 30 d: smoked cigarettes or E-cigarettes	301	3.1 (1.1–8.4)	0.029	1.7 (0.7–4.0)	0.245	1.4 (0.6–3.2)	0.448
Past 30 d: used marijuana	301	6.1 (1.8–20.6)	0.004	1.2 (0.5–2.7)	0.691	0.6 (0.3–1.3)	0.179
Lifetime: used 1 or more illicit drugs or prescription drugs without prescription	299	3.5 (1.5–8.3)	0.004	1.3 (0.6–2.7)	0.438	0.7 (0.4–1.5)	0.373
Past 12 m: someone offered, sold, or gave you an illegal drug on school property (in teens attending school)	264	3.0 (1.3–6.9)	0.010	0.9 (0.4–1.8)	0.708	0.7 (0.3–1.4)	0.288

Bullying and conflicts							
Past 12 m: in physical fight	301	0.6 (0.3–1.2)	0.157	1.1 (0.6–2.1)	0.812	0.7 (0.4–1.4)	0.304
Past 12 m: bullied at school (in teens attending school)	265	0.5 (0.2–1.1)	0.091	0.8 (0.4–1.8)	0.624	2.9 (1.2–6.8)	0.018
Past 12 m: electronically bullied	301	1.3 (0.5–3.5)	0.577	0.4 (0.1–0.9)	0.040	3.2 (1.1–9.4)	0.030

Depression and suicide-related behaviors							
Past 12 m: depressed ≥2 wk	301	0.8 (0.4–1.3)	0.334	0.4 (0.2–0.7)	0.001	0.6 (0.3–1.0)	0.063
Past 12 m: seriously considered attempting suicide	301	1.3 (0.6–2.9)	0.468	0.5 (0.2–1.0)	0.062	0.7 (0.4–1.6)	0.438
Past 12 m: made a suicidal plan	301	1.2 (0.5–2.8)	0.668	0.4 (0.2–0.9)	0.030	1.2 (0.5–2.8)	0.662
Past 12 m: attempted suicide^a^	299	0.7 (0.2–2.5)	0.611	0.3 (0.1–1.1)	0.064	0.6 (0.2–2.1)	0.454
Past 12 m: suicide attempt resulted in injury needing doctor or nurse (in teens who attempted suicide)^b^	11	—	—	—	—	—	—

^a^Odds ratios are unadjusted because the number of teens who had the behavior was too small for logistic regression. ^b^Statistics were not performed because the sample size is too small; 4/11 (36.4%) teens who attempted suicide required medical attention. ^c^Binge drinking is defined as having 5 or more drinks of alcohol in a row, within a couple of hours. AOR, adjusted odds ratio; CI, confidence interval; d, days; m, months; Ref., reference category; wk, weeks; y, years. Multiple logistic regression models included age category, gender, race, type of kidney disease, and number of medications/day. na, odds ratio not available because no 13- to 15-year-olds drank or used drugs the last time had sex.

**Table 4:** Ages (years) for first had sex, first had a drink, and first smoked marijuana.

Variable	Statistic	ASK KIDD	Control	*P* value
Age (years) first had sex	Mean ± SD	15.1 ± 1.6	14.6 ± 1.6	0.010
	Median (IQR)	15.0 (2.0)	15.0 (2.0)	
	Range	11–17	11–17	
	25th	14.0	14.0	
	75th	16.0	16.0	
	*n*	80	3189	

Age (years) had first drink	Mean ± SD	14.1 ± 2.3	13.3 ± 2.6	0.007
	Median (IQR)	15.5 (2.0)	13.5 (4.0)	
	Range	8–17	8–17	
	25th	13.5	11.5	
	75th	15.5	15.5	
	*n*	70	4805	

Age (years) first smoked marijuana	Mean ± SD	13.8 ± 2.4	13.8 ± 2.1	0.988
	Median (IQR)	13.5 (2.0)	13.5 (2.0)	
	Range	8–17	8–17	
	25th	13.5	13.5	
	75th	15.5	15.5	
	*n*	64	3282	

IQR = interquartile range.

**Table 5:** Attitudes towards alcohol and substance use.

Question	*n* (%)
How much do you think people risk harming themselves if they …	
Smoke one or more packs of cigarettes per day	
No risk	38 (12.6)
Slight risk	48 (15.9)
Moderate risk	57 (18.9)
Great risk	159 (52.6)
Take one or two drinks of an alcoholic beverage per day	
No risk	47 (15.6)
Slight risk	57 (18.9)
Moderate risk	105 (34.8)
Great risk	93 (30.8)
Smoke marijuana once or twice a week	
No risk	81 (26.8)
Slight risk	70 (23.2)
Moderate risk	69 (22.8)
Great risk	82 (27.2)
Use prescription drugs that are not prescribed to them	
No risk	38 (12.6)
Slight risk	48 (15.9)
Moderate risk	57 (18.9)
Great risk	159 (52.6)

*N* = 302 for all questions.

## Data Availability

The SPSS data file containing all subjects' raw data used to support the findings of this study is available from the corresponding author upon request.

## Supplementary Materials

Supplementary Materials Supplement 1: survey questions. Supplement 2: table of sample sizes in each of 8 strata for the ASK KIDD and YRBS data sets.
