# Supplementary material for: Risk Behaviors in Teens with Chronic Kidney Disease: A Study from the Midwest Pediatric Nephrology Consortium
Source: Int J Nephrol. 2019 Dec 4;2019:7828406. doi: 10.1155/2019/7828406 (PMC6914908; doi:10.1155/2019/7828406)
Supplement: Supplementary Materials — Supplement 1: survey questions. Supplement 2: table of sample sizes in each of 8 strata for the ASK KIDD and YRBS data sets. [file 7828406.f1.pdf]

Health Behavior Survey in Teens Who Have a Kidney Condition

Health Behavior Survey in Teens Who Have a Kidney Condition This survey is about health behavior. It has been developed so you can tell us what you do that may affect your health. Your responses are anonymous - no one will know what your answers are to any questions. The survey contains 46 questions. Make sure to read every question. If you're not sure of an answer, give your best guess. The survey will not allow you to skip any questions.

1. How old are you?
- ❍ 13 years old (1)
- ❍ 14 years old (2)
- ❍ 15 years old (3)
- ❍ 16 years old (4)
- ❍ 17 years old (5)
- ❍ 18 years old (6)
- ❍ 19 years old (7)

2. What is your sex?
- ❍ Female (1)
- ❍ Male (2)

3. What is your race?
- ❍ Black or African American (1)
- ❍ White (2)
- ❍ Other (3)

4. Which of the following best describes why you see a kidney doctor?
- ❍ I have a kidney transplant (1)
- ❍ I am on hemodialysis or peritoneal dialysis (2)
- ❍ I have a kidney condition or disease (3)

4a. How long have you had this kidney transplant? (show if Q4 = 1)
- ❍ Less than 1 year (1)
- ❍ 1-2 years (2)
- ❍ More than 2 years (3)

4b. What type of dialysis are you on? (show if Q4 = 2)
- ❍ Hemodialysis (1)
- ❍ Peritoneal dialysis (2)

4c. How long have you been on dialysis? (show if Q4 = 2)
- ❍ Less than 1 year (1)
- ❍ 1-2 years (2)
- ❍ More than 2 years (3)

5. How many regular medications do you take each day (the total number of pills, capsules, liquids, or shots each day)?
- ❍ None (1)
- ❍ 1-5 (2)
- ❍ 6-10 (3)
- ❍ 11-20 (4)
- ❍ More than 20 (5)

The next 4 questions ask about car or other vehicle safety:

6. How often do you wear a seat belt when riding in a car driven by someone else?
- ❍ Never (1)
- ❍ Rarely (2)
- ❍ Sometimes (3)
- ❍ Most of the time (4)
- ❍ Always (5)

7. During the past 30 days, how many times did you ride in a car or other vehicle driven by someone who had been drinking alcohol?
- ❍ 0 times (1)
- ❍ 1 time (2)
- ❍ 2 or 3 times (3)
- ❍ 4 or 5 times (4)
- ❍ 6 or more times (5)

8. During the past 30 days, how many times did you drive a car or other vehicle when you had been drinking alcohol?
- ❍ I did not drive a car or other vehicle during the past 30 days (1)
- ❍ 0 times (2)
- ❍ 1 time (3)
- ❍ 2 or 3 times (4)
- ❍ 4 or 5 times (5)
- ❍ 6 or more times (6)

9. During the past 30 days, on how many days did you text or e-mail while driving a car or other vehicle?
- ❍ I did not drive a car or other vehicle during the past 30 days (1)
- ❍ 0 days (2)
- ❍ 1 or 2 days (3)
- ❍ 3 to 5 days (4)
- ❍ 6 to 9 days (5)
- ❍ 10 to 19 days (6)
- ❍ 20 to 29 days (7)
- ❍ All 30 days (8)

The next 3 questions ask about physical fighting and bullying:

10. During the past 12 months, how many times were you in a physical fight?
- ❍ 0 times (1)
- ❍ 1 time (2)
- ❍ 2 or 3 times (3)
- ❍ 4 or 5 times (4)
- ❍ 6 or 7 times (5)
- ❍ 8 or 9 times (6)
- ❍ 10 or 11 times (7)
- ❍ 12 or more times (8)

11. During the past 12 months, have you ever been bullied on school property?
- ❍ No (1)
- ❍ Yes (2)
- ❍ I have graduated from school (3)
- ❍ I am home schooled (4)

12. During the past 12 months, have you ever been electronically bullied? (Include being bullied through email, chat rooms, instant messaging, websites, or texting.)
- ❍ No (1)
- ❍ Yes (2)

The next 5 questions ask about sad feelings and attempted suicide. Sometimes people feel so depressed about the future that they may consider attempting suicide, or taking some action to end their own life:

13. During the past 12 months, did you ever feel so sad or hopeless almost every day for two weeks or more in a row that you stopped doing some usual activities?
- ❍ No (1)
- ❍ Yes (2)

14. During the past 12 months, did you ever seriously consider attempting suicide?
- ❍ No (1)
- ❍ Yes (2)

15. During the past 12 months, did you make a plan about how you would attempt suicide?
- ❍ No (1)
- ❍ Yes (2)

16. During the past 12 months, how many times did you actually attempt suicide?
- ❍ 0 times (1)
- ❍ 1 time (2)
- ❍ 2 or 3 times (3)
- ❍ 4 or 5 times (4)
- ❍ 6 or more times (5)

17. If you attempted suicide during the past 12 months, did any attempt result in an injury, poisoning, or overdose that had to be treated by a doctor or nurse?
- ❍ I did not attempt suicide during the past 12 months (3)
- ❍ No (1)
- ❍ Yes (2)

The next 4 questions ask about drinking alcohol. This includes drinking beer, wine, wine coolers, and liquor such as rum, gin, vodka, or whiskey. For these questions, drinking alcohol does not include drinking a few sips of wine for religious purposes:

18. During your life, on how many days have you had at least one drink of alcohol?
- ❍ 0 days (1)
- ❍ 1 or 2 days (2)
- ❍ 3 to 9 days (3)
- ❍ 10 to 19 days (4)
- ❍ 20 to 39 days (5)

- 40 to 99 days (6)
- 100 or more days (7)

19. How old were you when you had your first drink of alcohol other than a few sips?
- I have never had a drink of alcohol other than a few sips (1)
- 8 years old or younger (2)
- 9 or 10 years old (3)
- 11 or 12 years old (4)
- 13 or 14 years old (5)
- 15 or 16 years old (6)
- 17 years old or older (7)

20. During the past 30 days, on how many days did you have at least one drink of alcohol?
- 0 days (1)
- 1 or 2 days (2)
- 3 to 5 days (3)
- 6 to 9 days (4)
- 10 to 19 days (5)
- 20 to 29 days (6)
- All 30 days (7)

21. During the past 30 days, on how many days did you have 5 or more drinks of alcohol in a row, that is, within a couple of hours?
- 0 days (1)
- 1 day (2)
- 2 days (3)
- 3 to 5 days (4)
- 6 to 9 days (5)
- 10 to 19 days (6)
- 20 or more days (7)

The next 3 questions ask about cigarette smoking and electronic cigarette (E-cigarette) use:

22. During the past 30 days, on how many days did you smoke cigarettes?
- 0 days (1)
- 1 or 2 days (2)
- 3 to 5 days (3)
- 6 to 9 days (4)
- 10 to 19 days (5)
- 20-29 days (6)
- All 30 days (7)

23. During the past 30 days, on the days you smoked, how many cigarettes did you smoke per day?
- I did not smoke cigarettes during the past 30 days (1)
- Less than 1 cigarette per day (2)
- 1 cigarette per day (3)
- 2 to 5 cigarettes per day (4)
- 6-10 cigarettes per day (5)
- 11-20 cigarettes per day (6)
- More than 20 cigarettes per day (7)

24. During the past 30 days, on how many days did you use electronic cigarettes (E-cigarettes)?
- ❍ 0 days (1)
- ❍ 1 or 2 days (2)
- ❍ 3 to 5 days (3)
- ❍ 6 to 9 days (4)
- ❍ 10-19 days (5)
- ❍ 20-29 days (6)
- ❍ All 30 days (7)

The next 3 questions ask about marijuana use. Marijuana also is called grass or pot:

25. During your life, how many times have you used marijuana?
- ❍ 0 times (1)
- ❍ 1 or 2 times (2)
- ❍ 3 to 9 times (3)
- ❍ 10 to 19 times (4)
- ❍ 20 to 39 times (5)
- ❍ 40 to 99 times (6)
- ❍ 100 or more times (7)

26. How old were you when you tried marijuana for the first time?
- ❍ I have never tried marijuana (1)
- ❍ 8 years old or younger (2)
- ❍ 9 or 10 years old (3)
- ❍ 11 or 12 years old (4)
- ❍ 13 or 14 years old (5)
- ❍ 15 or 16 years old (6)
- ❍ 17 years old or older (7)

27. During the past 30 days, how many times did you use marijuana?
- ❍ 0 times (1)
- ❍ 1 or 2 times (2)
- ❍ 3 to 9 times (3)
- ❍ 10 to 19 times (4)
- ❍ 20 to 39 times (5)
- ❍ 40 or more times (6)

The next 9 questions ask about drugs and other substances: During your life, how many times have you done any of the following?

	0 times	1 or 2 times	3 to 9 times	10-19 times	20-39 times	40+ times
28. Used cocaine, including powder, crack, or freebase?	❍	❍	❍	❍	❍	❍
29. Used heroin (also called smack, junk, or China White)?	❍	❍	❍	❍	❍	❍
30. Used methamphetamines (also called speed, crystal, crank, or ice)?	❍	❍	❍	❍	❍	❍
31. Used hallucinogenic drugs, such as LSD,acid, PCP, angel dust, mescaline, or mushrooms?)	❍	❍	❍	❍	❍	❍
32. Used ecstasy (also called MDMA)?	❍	❍	❍	❍	❍	❍
33. Used synthetic marijuana (also called K2, Spice, fake weed, King Kong, Yucatan Fire, Skunk, or Moon Rocks)?	❍	❍	❍	❍	❍	❍
34. Sniffed glue, breathed contents of aerosol spray cans, or inhaled any paints or sprays to get high?	❍	❍	❍	❍	❍	❍
35. Taken steroid pills or shots without a doctor's prescription?	❍	❍	❍	❍	❍	❍
36. Taken a prescription drug (such as OxyContin, Percocet, Vicodin, codeine, Adderall, Ritalin, or Xanax) without a doctor's prescription?	❍	❍	❍	❍	❍	❍

37. During the past 12 months, has anyone offered, sold, or given you an illegal drug on school property?

❍ No (1)
❍ Yes (2)
❍ I have graduated from school (3)
❍ I am home schooled (4)

The next 5 questions ask about sexual behavior:

38. How old were you when you had sexual intercourse for the first time?
- ❍ I have never had sexual intercourse (1)
- ❍ 11 years old or younger (2)
- ❍ 12 years old (3)
- ❍ 13 years old (4)
- ❍ 14 years old (5)
- ❍ 15 years old (6)
- ❍ 16 years old (7)
- ❍ 17 years old or older (8)

39. During the past 3 months, with how many people did you have sexual intercourse?
- ❍ I have never had sexual intercourse (1)
- ❍ I have had intercourse, but not during the past 3 months (8)
- ❍ 1 person (2)
- ❍ 2 people (3)
- ❍ 3 people (4)
- ❍ 4 people (5)
- ❍ 5 people (6)
- ❍ 6 or more people (7)

40. Did you drink alcohol or use drugs before you had sexual intercourse the last time?
- ❍ I have never had sexual intercourse (1)
- ❍ No (2)
- ❍ Yes (3)

41. The last time you had sexual intercourse, did you or your partner use a condom?
- ❍ I have never had sexual intercourse (1)
- ❍ No (2)
- ❍ Yes (3)

42. The last time you had sexual intercourse, what one method did you or your partner use to prevent pregnancy? (Select only one response.)
- ❍ I have never had sexual intercourse (1)
- ❍ No method used to prevent pregnancy (2)
- ❍ Birth control pills (3)
- ❍ Condoms (4)
- ❍ Depo-Provera (injectable birth control), Nuva Ring (or any birth control ring), Implanon (or any implant), or any IUD (5)
- ❍ Withdrawal (6)
- ❍ Some other method (7)
- ❍ Not sure (8)

The next 4 questions ask about attitudes toward tobacco, alcohol, marijuana, and prescription drugs:

43. How much do you think people risk harming themselves physically or in other ways if they smoke one or more packs of cigarettes per day?

- No risk (1)
- Slight risk (2)
- Moderate risk (3)
- Great risk (4)

44. How much do you think people risk harming themselves physically or in other ways if they take one or two drinks of an alcoholic beverage nearly every day?

- No risk (1)
- Slight risk (2)
- Moderate risk (3)
- Great risk (4)

45. How much do you think people risk harming themselves physically or in other ways if they smoke marijuana once or twice a week?

- No risk (1)
- Slight risk (2)
- Moderate risk (3)
- Great risk (4)

46. How much do you think people risk harming themselves physically or in other ways if they use prescription drugs that are not prescribed to them?

- No risk (1)
- Slight risk (2)
- Moderate risk (3)
- Great risk (4)

Supplement 2

Comparisons between ASKKIDD proportions and hypothesized population proportions: the YRBS 2015 proportions were stratified by age,gender, and race and then randomly sampled within each stratum to obtain an overall sample that exactly matched the ASKKIDD proportions.

Age (years), sex, and race stratum	ASKKIDD No. (%)	2015 Natl. YRBS stratified random sample No. (%)	2015 Natl. YRBS entire sample* No. (%)
13-15, female, white	26 (8.4)	718 (8.4)	1363 (8.9)
13-15, female, nonwhite	24 (7.8)	663 (7.8)	1461 (9.6)
13-15, male, white	43 (13.9)	1186 (13.9)	1186 (7.8)
13-15, male, nonwhite	24 (7.8)	663 (7.8)	1434 (9.4)
16-19, female, white	40 (12.9)	1104 (12.9)	2123 (13.9)
16-19, female, nonwhite	43 (13.9)	1186 (13.9)	2719 (17.8)
16-19, male, white	65 (21.0)	1795 (21.0)	2220 (14.5)
16-19, male, nonwhite	44 (14.2)	1215 (14.2)	2788 (18.2)
Total	309 (100.0)	8530 (100.0)	15294 (100.0)

*Of 15624 respondents, 43 were 12 years old and were excluded, and 287 were missing age, gender, and/or race.
